# Efficacy of combination chemotherapy using a novel oral chemotherapeutic agent, TAS-102, together with bevacizumab, cetuximab, or panitumumab on human colorectal cancer xenografts

**DOI:** 10.3892/or.2015.3876

**Published:** 2015-03-23

**Authors:** HIROSHI TSUKIHARA, FUMIO NAKAGAWA, KAZUKI SAKAMOTO, KEIJI ISHIDA, NOZOMU TANAKA, HIROYUKI OKABE, JUNJI UCHIDA, KENICHI MATSUO, TEIJI TAKECHI

**Affiliations:** 1 Translational Research Laboratory, Tokushima Research Center, Taiho Pharmaceutical Co., Ltd., Kawauchi-Cho, Tokushima-shi, Tokushima 771-0194, Japan; 2 Applied Pharmacology Laboratory, Tokushima Research Center, Taiho Pharmaceutical Co., Ltd., Kawauchi-Cho, Tokushima-shi, Tokushima 771-0194, Japan; 3 Tsukuba Research Center, Taiho Pharmaceutical Co., Ltd., Okubo, Tsukuba, Ibaraki 300-2611, Japan

**Keywords:** colorectal cancer, tipiracil hydrochloride bevacizumab, TAS-102, trifluridine, cetuximab, panitumumab

## Abstract

TAS-102 is a novel oral nucleoside antitumor agent that consists of trifluridine (FTD) and tipiracil hydrochloride (TPI) at a molecular ratio of 1:0.5, and was approved in Japan in March 2014 for the treatment of patients with unresectable advanced or recurrent colorectal cancer that is refractory to standard therapies. In the present study, we used colorectal cancer xenografts to assess whether the efficacy of TAS-102 could be improved by combining it with bevacizumab, cetuximab or panitumumab. TAS-102 was orally administered twice a day from day 1 to 14, and bevacizumab, cetuximab and panitumumab were administered intraperitoneally twice a week for 2 weeks. Growth inhibitory activity was evaluated based on the relative tumor volume (RTV) after 2 weeks of drug administration and time taken for the relative tumor volume to increase five-fold (RTV5). Tumor growth inhibition and RTV5 with TAS-102 and bevacizumab combination treatment were significantly better than those with TAS-102 or bevacizumab alone in the SW48 and HCT116 tumor models, and the concentration of phosphorylated FTD in tumors determined by liquid chromatography-tandem mass spectrometry (LC-MS/MS) analysis was higher in the TAS-102 and bevacizumab combination group than in the TAS-102 monotherapy group. The combination of TAS-102 and cetuximab or panitumumab was also significantly more effective than either monotherapy in the SW48 tumor model. There was no significant difference in the body weight between the mice treated with TAS-102 monotherapy and any of the combination therapies on day 29. Our preclinical findings indicate that the combination therapy of TAS-102, bevacizumab and cetuximab or panitumumab is a promising treatment option for colorectal cancer.

## Introduction

Worldwide, colorectal cancer is the third most common cancer (9.7%) and it was the fourth leading cause of cancer-related mortality in 2012 (1). For the treatment of unresectable metastatic colorectal cancer, systemic chemotherapeutic agents such as fluoropyrimidines, irinotecan (CPT-11), oxaliplatin, and targeted agents such as bevacizumab (an anti-VEGF monoclonal antibody) and cetuximab, or panitumumab (anti-EGFR monoclonal antibodies) are currently used, while the survival of patients with unresectable metastatic colorectal cancer has improved (2–5). Even if these standard therapies are initially effective, many patients relapse due to the onset of drug resistance and are subsequently placed on salvage chemotherapy. The multikinase inhibitor regorafenib was reported to prolong the overall survival compared to placebo for the treatment of unresectable refractory colorectal cancer (6).

TAS-102 is a combination of an antineoplastic thymidine-based nucleoside analogue, trifluridine (FTD) and a thymidine phosphorylase inhibitor, tipiracil hydrochloride (TPI) at a molecular ratio of 1:0.5. FTD is the active antitumor component of TAS-102; its monophosphate form inhibits thymidylate synthase, and its triphosphate form is incorporated into the DNA in tumor cells. The inhibition of thymidylate synthase caused by oral FTD rapidly disappears after the drug elimination, but the incorporation of FTD into the DNA is known to have prolonged antitumor effects (7–9).

When FTD is administered orally, it is rapidly degraded to its inactive form in the intestines and the liver (first-pass effect) (8), but the combination with TPI helps to maintain adequate FTD plasma concentrations (10). TPI thus, potentiates the antitumor activity of FTD (10), and the optimal molecular ratio of FTD to TPI has been proven to be 1:0.5 (11). In preclinical studies, both FTD and TAS-102 were found to exhibit some unique antitumor effects, such as their efficacy against 5-FU-resistant colorectal tumor cells not only *in vitro* but also *in vivo* (12–14), and a continued effect persisted after the end of drug administration (9,15).

In a randomized phase II trial, the overall survival period of patients receiving TAS-102 with the best supportive care (9 months) was significantly longer than that of a placebo with the best supportive care group (6.6 months, P=0.0011) in patients with metastatic colorectal cancer, who were refractory to or intolerant of standard chemotherapies (16). TAS-102 showed a significant improvement in overall and progression free survival and a favorable safety profile in comparison to placebo in patients with metastatic colorectal cancer refractory to standard chemotherapies in an international multicenter randomized double-blind phase III study (RECOURSE), patients received in both arms the best supportive care (17). TAS-102 was approved for clinical use in Japan in March 2014. Bevacizumab and cetuximab or panitumumab are key drugs in colorectal cancer treatment, used either alone or in combination with other chemotherapies (3–5,18–21).

In the present study, we evaluated the antitumor effects of TAS-102 in combination with bevacizumab and cetuximab or panitumumab using a nude mouse xenograft model of colorectal cancer.

## Materials and methods

### Reagents

FTD, F_3_TMP ammonium salt, F_3_TDP, F_3_TTP and TPI were obtained from Taiho Pharmaceutical (Tokyo, Japan). Bevacizumab and cetuximab or panitumumab were purchased from Roche (Basel, Switzerland), Merck Serono (Darmstadt, Germany), and Amgen (Thousand Oaks, CA, USA), respectively. Hydroxypropyl methylcellulose (HPMC) was purchased from Shin-Etsu Chemical (Tokyo, Japan).

### Cancer cell lines

The human colon cancer cell lines SW48 and HCT116 were purchased from the American Type Culture Collection (ATCC; Rockville, MD, USA), and Dainippon Pharma (Osaka, Japan), respectively. SW48 and HCT116 cells were maintained by implantation into the right axilla of nude mice at 3-week intervals. The *KRAS* mutation status of SW48 and HCT116 are wild-type and mutant, respectively (22).

### Animals

Male nude mice were purchased from CLEA Japan (Tokyo, Japan) and were housed under specific pathogen-free conditions, with food and water provided *ad libitum*. All the animal studies were performed according to the instructions and with the approval of the Institutional Animal Care and use Committee of Taiho Pharmaceutical Co. (approval nos. 14Tb04, M01-2008-0004, 03-12-008 and AM003-14-016).

### Antitumor activity in vivo

After the animals had been in quarantine for 1 week, they were implanted subcutaneously with a solid human tumor, the volume of which was ~8 mm^3^ (23). In order to evaluate the antitumor activity, the mice were randomized on day 0 according to tumor volume, once the mean tumor volume had reached ~100–200 mm^3^. Each group consisted of 6 or 7 mice.

TAS-102 was prepared by mixing FTD and TPI in a molecular ratio of 1:0.5 in 0.5% HPMC solution. The dose of TAS-102 was expressed on the basis of the amount of FTD, and was administered orally from day 1 to 14, twice a day at ~6-h intervals at the reported effective dose (150 mg/kg/day) (7,11). For the control group, vehicle (0.5% HPMC solution) was administered at 10 ml/kg in a similar manner. Bevacizumab was administered intraperitoneally in a dose of 5 mg/kg on days 1, 4, 8 and 11. Cetuximab and panitumumab were administered intraperitoneally in a dose of 4.4 and 3 mg/kg, respectively, on days 1, 5, 8 and 12.

Tumor diameters were measured twice a week, and the tumor volume was estimated as 0.5 × length × width^2^. The relative tumor volume (RTV) was calculated using the following formula: RTV = (tumor volume on measured day)/(tumor volume on day 0). On day 29, the tumor growth inhibition ratio (TGI, %) was calculated using the following formula: TGI (%) = [1 − (RTV of the treated group)/(RTV of the control group)] × 100 (%).

Antitumor activity was evaluated on the basis of the time taken for the relative tumor volume to increase five-fold (RTV5). In order to assess RTV5, the RTV change of each mouse was plotted and the date when RTV5 was reached was estimated using linear regression based on the dates on either side of this event (24).

To evaluate toxicity, body weight was measured twice a week and body weight change (BWC) was calculated using the following formula: BWC (%) = [(body weight on the last day) − (body weight on day 0)]/(body weight on day 0) × 100 (%). Toxicity was defined as a BWC of <−20%, or toxic mortality.

### Extraction and quantification of tumor FTD and its phosphorylated forms

FTD and its phosphorylated forms were determined by liquid chromatograph-mass spectrometry (LCMS-8040; Shimadzu, kyoto, Japan). TAS-102 was administered orally from day 1 to 3 twice a day (150 mg/kg) and bevacizumab was administered on day 1 (5 mg/kg) into nude mice bearing SW48 and HCT116 xenografts. Each group consisted of 5 mice. Two hours after the last TAS-102 administration, mice were sacrificed and tumors were collected and frozen quickly by using liquid nitrogen.

For extraction of FTD and its metabolite, the tumors were homogenized in 0.48 N perchloric acid solution with a Multi-Beads Shocker (Yasui Kikai, Osaka, Japan), and centrifuged at 20,000 × g for 5 min at 4°C. The aqueous phase was recovered and, twice the volume of the mixture of 0.5 N tri-n-octylamine and dichloromethane (1:3) was added to the acid soluble fractions and mixed by vortexing. Then samples were centrifuged at 20,000 × g for 5 min at 4°C. The aqueous phases were collected and used as samples for the next mass spectrometric analysis. Samples (5 *μ*l) were analyzed on a triple quadruple mass spectrometer (LCMS-8040; Shimadzu), with a Mastro C18 column (3 *μ*m particle size, length 150 mm and inner diameter 2.1 mm; Shimadzu GLC, Tokyo, Japan). Samples from xenografts which were not administrated TAS-102 were used as blank samples. FTD, F_3_TMP ammonium salt, F_3_TDP and F_3_TTP were mixed at an equally molecular ratio and a standard solution was prepared at the concentration of 10, 3, 1, 0.3, 0.1 0.03 and 0.01 *μ*M for each compound. The mobile phase consisted of a linear gradient of 0.5 mM dibutylammonium acetate in distilled water (A) 100% methanol (B): 0–4 min, 1–60% B (v/v); 4–10 min, 60–60% B; 10–10.1 min, 60–1% B; 10.1–21 min, 1–1% B. The flow rate was 0.2 ml/min. The effluent from the column was measured by mass spectrometry using electrospray ionization (ESI). ESI parameters were as follows: interface temperature 350°C, gas flow 3 l/min, heat-block temperature 400°C, and drying gas flow 15 l/min. The mass spectrometer was operated in the negative ion mode using LabSolution software version 5.60 SP2 (Shimadzu) in a multiple reaction monitoring mode. The monitored transitions were m/z 295.05>179.25 for FTD, m/z 375.05>179.20 for F_3_dTMP, m/z 454.95>275.05 for F_3_dTDP, and m/z 534.95>159.10 for F_3_dTTP. The lower limit of quantification (LLOQ) was set up as a signal to noise ratio of 3 by analyzing the standard tumor lysate. The LLOQs of FTD, F_3_dTMP, F_3_dTDP and F_3_dTTP were 0.18, 0.06, 0.06, and 1.8 nmol/g tissue in SW48 lysate, and 0.06, 0.06, 0.06, and 0.6 nmol/g tissue in HCT116 lysate, respectively. Values of FTD phosphates were calculated by taking the sum of F_3_dTMP, F_3_dTDP and F_3_dTTP for each mouse.

### Statistical analysis

The significance of the differences in the mean RTV between the treated and the control groups on day 29 was analyzed by using the Aspin-Welch two-sided t-test. The combinational antitumor effect of TAS-102 and bevacizumab, cetuximab or panitumumab was analyzed according to a closed-testing procedure using the Aspin-Welch two-tailed t-test (25). The statistical analysis of RTV5 was evaluated using the log-rank test according to the reported method (26). In cases where the RTV of the treated animal was not reached, the data were censored and the RTV5 was designated as 28 or 29. Differences with an associated P-value of <0.05 were considered significant. P-values were calculated using Exsus, version 8.1 (Arm Systex, Osaka, Japan).

The significance of increased FTD, F_3_dTMP, F_3_dTDP, and F_3_dTTP in the treated groups compared to the control groups was evaluated by using the Student’s one-sided t-test with statistical software JMP^®^, version 9.0.2 (SAS Institute, Cary, NC, USA).

## Results

### Bevacizumab increases the antitumor efficacy of TAS-102

**Figure 1:**
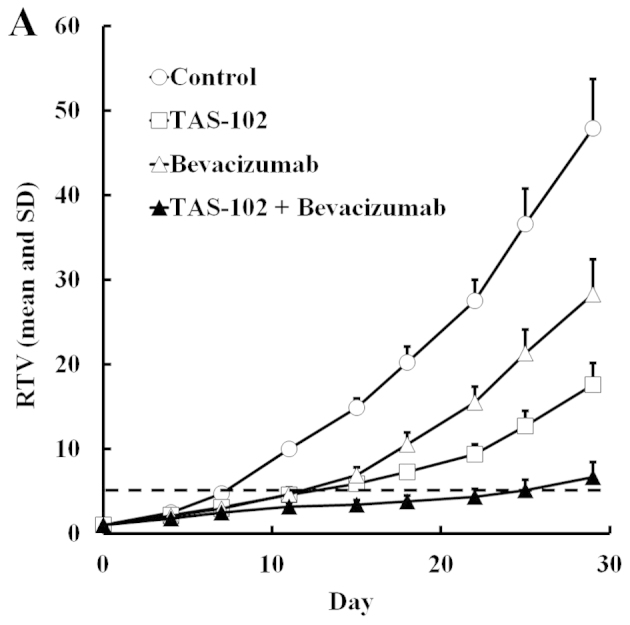

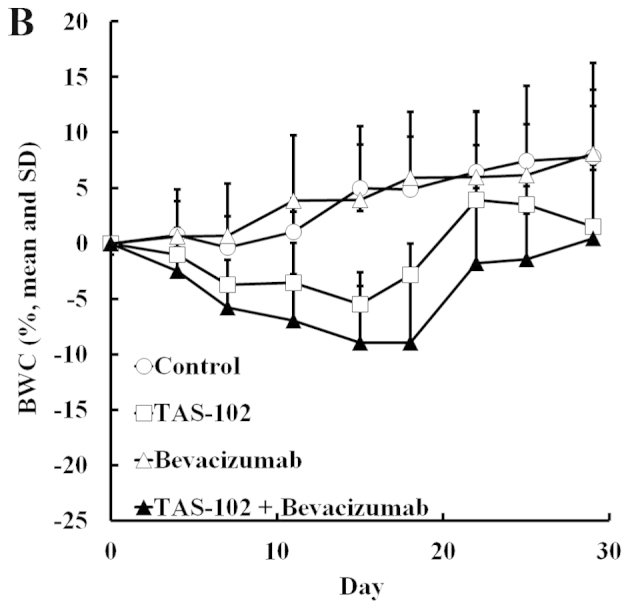
Relative volume change in human SW48 colorectal tumors (A), and body weight change in SW48 tumor-bearing nude mice (B). Mice were treated with vehicle (○), TAS-102 (□), bevacizumab (△), or combined TAS-102 and bevacizumab (▲). The values indicate the means + SD (n=6). The horizontal dotted line indicates an RTV of 5. RTV, relative tumor volume.

**Figure 2:**
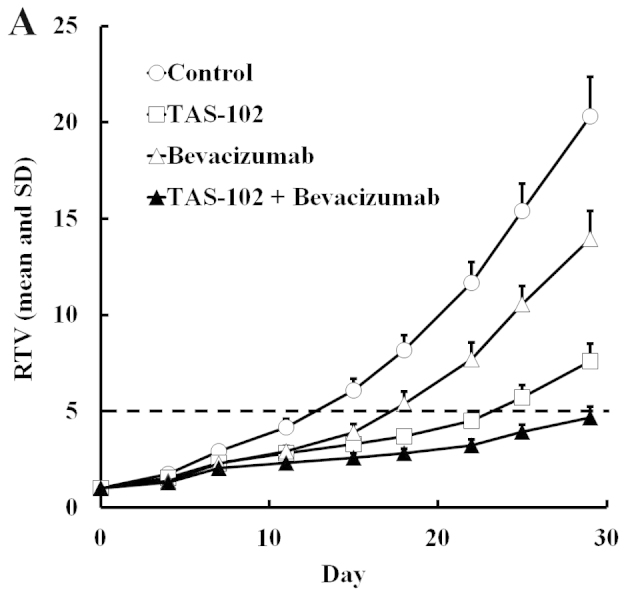

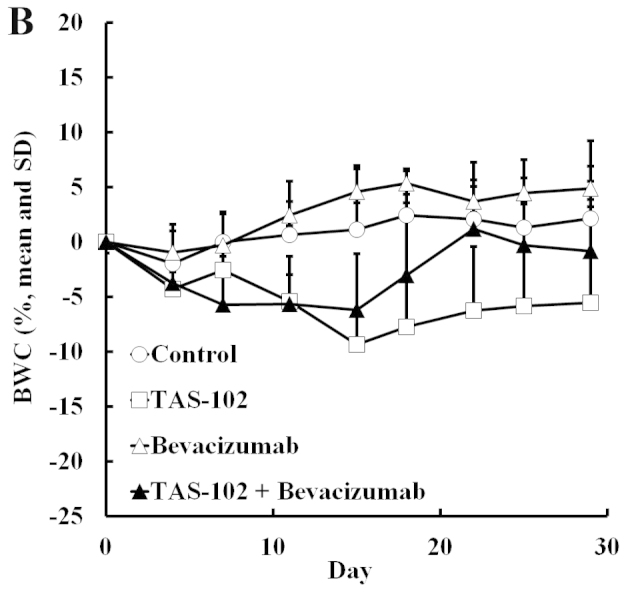
Relative volume change in human HCT116 colorectal tumors (A) and body weight change in HCT116 tumor-bearing nude mice (B). Mice were treated with vehicle (○), TAS-102 (□), bevacizumab (△), or combined TAS-102 and bevacizumab (▲). The values indicate the means + SD (n=6). The horizontal dotted line indicates an RTV of 5. RTV, relative tumor volume.

TAS-102 and bevacizumab either alone or in combination, were administered to mice bearing SW48 or HCT116 colorectal tumors. The RTV change and BWC in SW48 and HCT116 are shown in Figs. 1 and 2, respectively. In both experiments, TAS-102 and bevacizumab alone inhibited tumor growth. Moreover, combined TAS-102 and bevacizumab treatment had superior antitumor activity compared to either drug alone, and had no significant effect on the body weight compared to TAS-102 monotherapy.

We also evaluated the RTV5 of tumors. TAS-102 or bevacizumab alone significantly extended the RTV5 (P<0.01), but combined TAS-102 and bevacizumab extended the RTV5 still further relative to either monotherapy in both SW48 and HCT116 xenografts (Tables I and II). For SW48 tumors, the RTV5 of the combination group was more than twice as long as the bevacizumab monotherapy group, and for the HCT116 tumors, 4 of 6 mice treated with combination therapy did not reach RTV5 by day 29.

### Increased FTD and FTD phosphate tumor levels after being combined with bevacizumab and TAS-102 treatment

**Figure 3:**
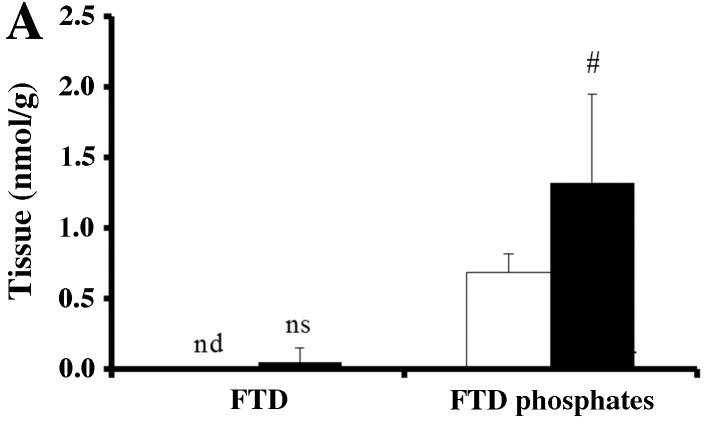

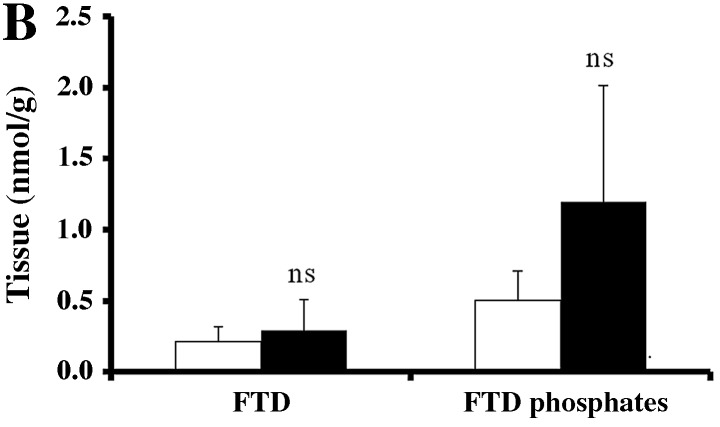
Concentration of FTD and its phosphorylated forms (F_3_dTMP, F_3_dTDP, and F_3_dTTP) in SW48 (A) and HCT116 (B) tumors administered TAS-102 alone (open bar, n=5) or in combination with TAS-102 and bevacizumab (closed bar, n=5) determined by LC-MS/MS analysis. Values are given as the mean ± SD. ^#^P<0.05 by the Student’s t-test compared to the TAS-102 group; ns, not significant; nd, not detected; LC-MS/MS, liquid chromatography-tandem mass spectrometry.

To investigate why bevacizumab improves the antitumor effect of TAS-102, we measured the concentration of FTD and its phosphates (F_3_dTMP, F_3_dTDP and F_3_dTTP) in SW48 and HCT116 tumors. Very little FTD was detected in SW48 tumors. FTD phosphates level was significantly higher in the TAS-102 and bevacizumab combination group in SW48 tumors compared to that from mice treated with TAS-102 monotherapy (P<0.05, Fig. 3A).

In HCT116 tumors, FTD was detected. Although it was not significant, FTD and FTD phosphates tended to increase after combined TAS-102 and bevacizumab treatment compared to TAS-102 monotherapy (Fig. 3b).

### Cetuximab and panitumumab increase the antitumor efficacy of TAS-102

**Figure 4:**
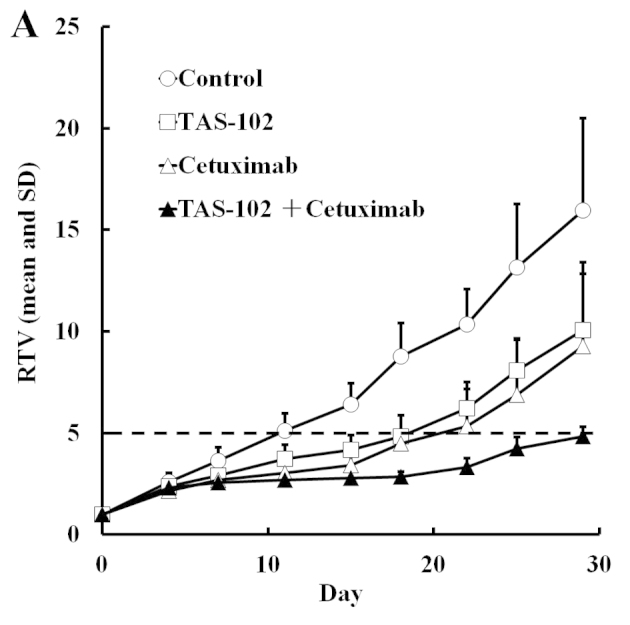

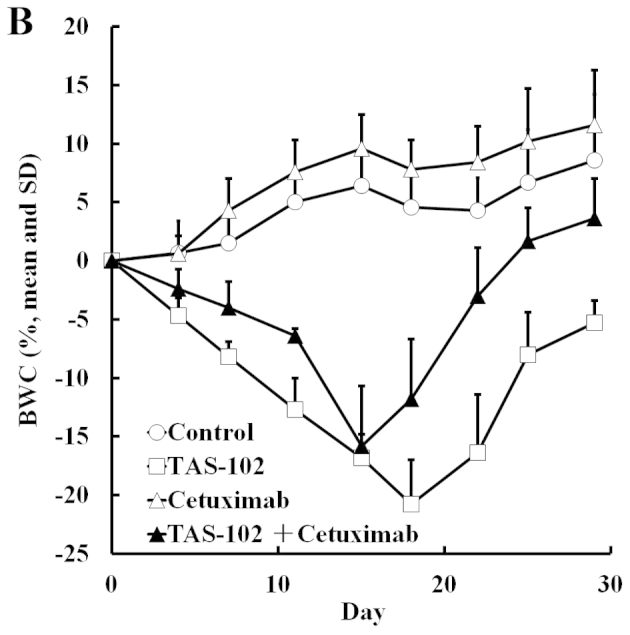
Relative volume change in human SW48 colorectal tumors (A) and body weight change in SW48 tumor-bearing nude mice (B). Mice were treated with vehicle (○), TAS-102 (□), cetuximab (△), or combined TAS-102 and cetuximab (▲). The values indicate the means + SD (n=6). The horizontal dotted line indicates an RTV of 5. RTV, relative tumor volume.

We evaluated the efficacy of cetuximab and panitumumab combined with TAS-102 in the SW48 xenograft model. TAS-102 and cetuximab both suppressed tumor growth compared to the vehicle alone (P<0.05 and 0.01, respectively, Table III), and combined cetuximab and TAS-102 significantly suppressed tumor growth compared to each monotherapy on day 29. Similarly, combined TAS-102 and cetuximab significantly extended the RTV5 compared to either drug alone. TAS-102 caused a significant reduction in the body weight compared to untreated mice (P<0.01) at the nadir on day 15 and 18, but the mice recovered and the weight loss was <10% on day 29. Thus, the toxicity of TAS-102 seemed to be tolerable (Fig. 4). Interestingly, combined cetuximab and TAS-102 did not result in significant body weight loss, despite having superior antitumor efficacy (Fig. 4).

**Figure 5:**
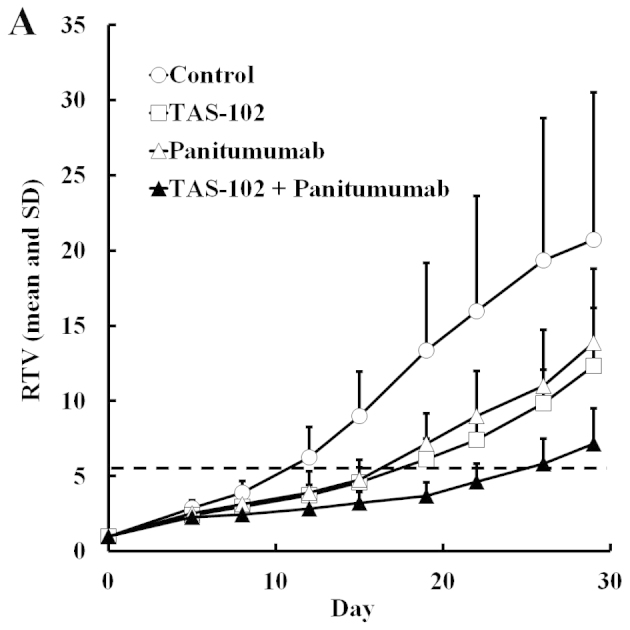

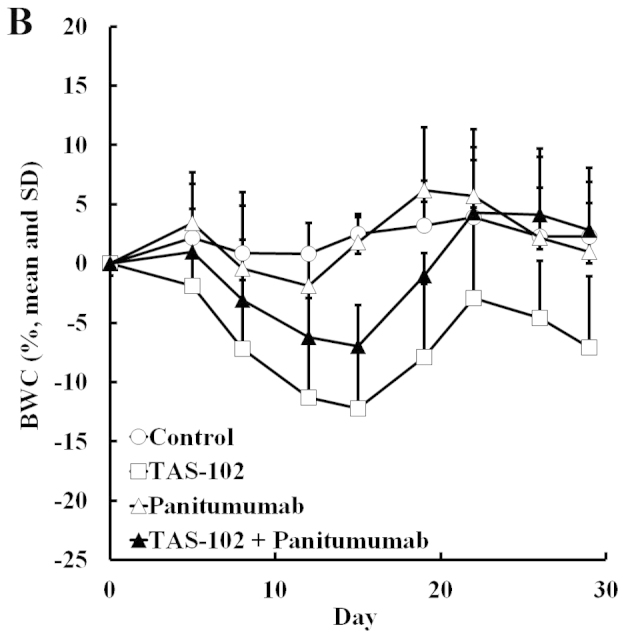
Relative volume change in human SW48 colorectal tumors (A) and body weight change in SW48 tumor-bearing nude mice (B). Mice were treated with vehicle (○), TAS-102 (□), panitumumab (△), or combined TAS-102 and panitumumab (▲). The values indicate the means + SD (n=6). The horizontal dotted line indicates an RTV of 5. RTV, relative tumor volume.

TAS-102 or panitumumab monotherapy tended to inhibit tumor growth but these reductions were not significant, since the standard deviation of RTV in the control group varied only in this experiment. Combined TAS-102 and panitumumab significantly reduced tumor volume and extended RTV5 (P<0.05 and 0.01, respectively, Table IV), while the combined therapy also resulted in less weight loss than TAS-102 alone, despite showing a superior antitumor effect (Fig. 5).

## Discussion

In the present study, we found that combined bevacizumab and TAS-102 suppresses tumor growth to a significantly greater degree than either drug alone in nude mice with colorectal cancer, but had no significant effect on the body weight. Thus, bevacizumab appears to enhance the antitumor effect of TAS-102 without increasing its toxicity.

We used two colorectal cancer cell lines: SW48, which is *KRAS* wild-type, and HCT116, which carries a *KRAS* mutation. TAS-102 was effective regardless of the *KRAS* status, at least in the present study. In a randomized phase-II trial for metastatic colorectal cancer patients who were refractory or intolerant to standard chemotherapies, TAS-102 also improved overall survival regardless of the *KRAS* tumor status (16). It has also been reported that the effect of bevacizumab is not influenced by the *KRAS* status (27,28). Furthermore, combined TAS-102 and bevacizumab showed superior antitumor efficacy to TAS-102 alone, and therefore, this combination therapy may be beneficial to patients with both mutated and wild-type *KRAS* tumors.

In order to evaluate the mechanism underlying the enhanced antitumor effect of combined TAS-102 and bevacizumab, we measured FTD and its phosphorylated forms in tumors, as these are the active components and metabolites of TAS-102. Phosphorylated FTD levels were increased by combining TAS-102 and bevacizumab in both SW48 and HCT116 tumors. Tumor blood vessels are generally poorly organized and hyperpermeable, with an impaired gradient between vascular and interstitial pressure and, consequently, a diminished blood supply (29). This may also limit the accumulation of FTD in tumors. Bevacizumab inhibits angiogenesis through antagonizing vascular endothelial growth factor and may therefore normalize tumor vasculature, improving tumor blood supply and increasing FTD accumulation and its subsequent phosphorylation in the tumor.

We also evaluated the combination of TAS-102 and the anti-epidermal growth factor receptor antibodies, cetuximab and panitumumab, in SW48 and *KRAS* wild-type tumors. Both enhanced the antitumor effect of TAS-102. Interestingly, combining TAS-102 with cetuximab or panitumumab reduced the weight loss that occurred after TAS-102 monotherapy. We observed no severe toxicity after combination treatment, as reflected by the absence of weight loss or drug-related deaths. However, other toxicities were not evaluated. In some clinical studies, most frequently observed toxicities were gastrointestinal and hematologic in phase II and III of TAS-102 (16,17). Careful monitoring of the overall side effects, including hematological toxicities, will be needed to evaluate the efficacy of these combination therapies in clinical studies.

In conclusion, we have demonstrated that bevacizumab, cetuximab and panitumumab enhance the antitumor effect of TAS-102 in colorectal cancer. These combination therapies may be proven to be promising options for patients suffering from cancer that is refractory to the existing drugs. A clinical study of combined TAS-102 and bevacizumab therapy is ongoing (no. UMIN000012883), and we expect that its outcome will be highly informative.

## Figures and Tables

**Table I:** Antitumor activity and body weight changes in mice implanted with human colorectal tumor SW48 after treatment with TAS-102 and bevacizumab.

Group	Dose (mg/kg)	Schedule	RTV^a^ (mean ± SD)	TGI^b^ (%)	RTV5^c^ (days)	BWC^d^	
						(Mean ± SD, g)	(%)
Control	–	–	47.94±5.78	0	7.23±0.23	2.0±2.0-	7.8
TAS-102	150	Day 1–14 (b.i.d.)	17.56±4.12^e^	63.4	12.49±2.66^g^	0.4±2.9 NS	1.5
Bevacizumab	5	Day 1, 4, 8, 11	28.27±2.61^e^	41.0	11.61±1.07^g^	2.1±1.5 NS	8.1
Combination	150+5		6.66±1.75^e^,^f^	86.1	24.72±4.24^g^,^h^	0.1±1.7 NS	0.4

a Relative tumor volume on day 29;

b Tumor growth inhibition ratio on day 29;

c The period, RTV reaches 5;

d Body weight change from day 0 to day 29; Each group consists of 6 mice;

e P<0.001 vs. control using the two-sided Aspin Welch t-test;

f P<0.001 by closed testing procedure using the two-sided Aspin-Welch t-test;

g P<0.001 vs. control using the log-rank test;

h P<0.001 vs. either monotherapy using the log-rank test; NS vs. control using the two-sided Aspin-Welch t-test; BWC, body weight change; RTV, relative tumor volume; TGI, tumor growth inhibition; NS, not significant.

**Table II:** Antitumor activity and body weight changes in mice implanted with human colorectal tumor HCT116 after treatment with TAS-102 and bevacizumab.

Group	Dose (mg/kg)	Schedule	RTV^a^ (mean ± SD)	TGI^b^ (%)	RTV5^c^ (days)	BWC^d^	
						(Mean ± SD, g)	(%)
Control	–	–	20.32±2.04	0	12.81±1.06	0.6±1.9-	2.2
TAS-102	150	Day 1–14 (b.i.d.)	7.60±0.90^e^	62.6	23.24±1.41^g^	−1.4±2.2 NS	−5.6
Bevacizumab	5	Day 1, 4, 8, 11	13.97±1.43^e^	31.3	17.32±1.17^g^	1.3±0.5 NS	4.9
Combination	150+5		4.66±0.58^e^,^f^	77.1	>28.57^g^,^h^	−0.2±1.6 NS	−0.8

a Relative tumor volume on day 29;

b Tumor growth inhibition ratio on day 29;

c The period, RTV reaches 5;

d Body weight change from day 0 to day 29; Each group consists of 6 mice;

e P<0.001 vs. control using the two-sided Aspin Welch t-test;

f P<0.001 by closed testing procedure using the two-sided Aspin-Welch t-test;

g P<0.001 vs. control using the log-rank test;

h P<0.001 vs. either monotherapy using the log-rank test; NS vs. control using the two-sided Aspin-Welch t-test; BWC, body weight change; RTV, relative tumor volume; TGI, tumor growth inhibition; NS, not significant.

**Table III:** Antitumor activity and body weight changes in mice implanted with human colorectal tumor SW48 after treatment with TAS-102 and cetuximab.

Group	Dose (mg/kg)	Schedule	RTV^a^ (mean ± SD)	TGI^b^ (%)	RTV5^c^ (days)	BWC^d^	
						(Mean ± SD, g)	(%)
Control	–	–	15.95±4.54	0	11.65±2.10	2.2±1.4-	8.6
TAS-102	150	Day 1–14 (b.i.d.)	10.05±3.22^f^	37.0	19.65±5.25^h^	−1.3±0.4^e^	−5.3
Cetuximab	4.4	Day 1, 5, 8, 12	9.29±2.79^e^	41.7	21.15±3.92^i^	3.0±1.2 NS	11.6
Combination	150+4.4		4.85±0.46^e^,^g^	69.6	>28.34^i^,^j^	0.9±0.8 NS	3.6

a Relative tumor volume on day 29;

b Tumor growth inhibition ratio on day 29;

c The period, RTV reaches 5;

d Body weight change from day 0 to day 29; Each group consists of 6 mice;

e P<0.01 and

f P<0.05, respectively vs. control using the two-sided Aspin Welch t-test;

g P<0.05 by closed testing procedure using the two-sided Aspin-Welch t-test;

h P<0.01 and

i P<0.001, respectively vs. control using the log-rank test;

j P<0.01 vs. either monotherapy using the log-rank test; NS vs. control using the two-sided Aspin-Welch t-test; BWC, body weight change; RTV, relative tumor volume; TGI, tumor growth inhibition; NS, not significant.

**Table IV:** Antitumor activity and body weight changes in mice implanted with human colorectal tumor SW48 after treatment with TAS-102 and panitumumab.

Group	Dose (mg/kg)	Schedule	RTV^a^ (mean ± SD)	TGI^b^ (%)	RTV5^c^ (days)	BWC^d^	
						(Mean ± SD, g)	(%)
Control	–	–	20.70±9.81	0	11.51±4.84	0.6±1.5-	2.3
TAS-102	150	Day 1–14 (b.i.d.)	12.33±3.86 NS	40.5	16.40±2.37 NS	−1.8±1.6^e^	−7.1
Panitumumab	3	Day 1, 5, 8, 12	13.86±4.94 NS	33.1	15.59±4.33 NS	0.3±1.1 NS	1.0
Combination	150+3		7.15±2.34^e^	65.5	>23.85^f^,^g^	0.7±1.0 NS	2.8

a Relative tumor volume on day 29;

b Tumor growth inhibition ratio on day 29;

c The period, RTV reaches 5;

d Body weight change from day 0 to day 29; Each group consists of 7 mice;

e P<0.05 vs. control using the two-sided Aspin Welch t-test;

f P<0.01 vs. control using the log-rank test;

g P<0.01 vs. either monotherapy using the log-rank test; NS vs. control; BWC, body weight change; RTV, relative tumor volume; TGI, tumor growth inhibition; NS, not significant.

## Abbreviations

BWC: body weight change
FTD: trifluridine
F_3_dTTP: α,α,α-trifluorothymidine triphosphate
HPMC: hydroxypropyl methylcellulose
LLOQ: lower limit of quantification
RTV: relative tumor volume
RTV5: time taken for the relative tumor volume to increase five-fold
TGI: tumor growth inhibition
LC-MS/MS: liquid chromatography-tandem mass spectrometry
TPI: tipiracil hydrochloride
F_3_dTMP: α,α,α-trifluorothymidine monophosphate
F_3_dTDP: α,α,α-trifluorothymidine diphosphate
